# Clinical indications and triaging for adult transthoracic echocardiography: a consensus statement by the British Society of Echocardiography in collaboration with British Heart Valve Society

**DOI:** 10.1186/s44156-022-00003-8

**Published:** 2022-07-13

**Authors:** Sadie Bennett, Martin Stout, Thomas E. Ingram, Keith Pearce, Timothy Griffiths, Simon Duckett, Grant Heatlie, Patrick Thompson, Judith Tweedie, Jo Sopala, Sarah Ritzmann, Kelly Victor, Judith Skipper, Benoy N. Shah, Shaun Robinson, Andrew Potter, Daniel X. Augustine, Claire L. Colebourn

**Affiliations:** 1 University Hospitals of North Midlands, Stoke-on-Trent, ST4 6QG UK; 2 Manchester University NHS Foundation Trust, Manchester, UK; 3 Shrewsbury and Telford Hospitals NHS Trust, Shrewsbury, UK; 4 Southern Health and Social Care Trust, Craigavon Area Hospital, Portadown, UK; 5 British Society of Echocardiography, London, UK; 6 Doncaster and Bassetlaw Teaching Hospitals, NHS Foundation Trust Doncaster Royal Infirmary, Doncaster, UK; 7 Cleveland Clinic, London, UK; 8 Barking, Havering and Redbridge University Hospitals NHS Trust, London, UK; 9 University Hospital Southampton NHS Foundation Trust, Southampton, UK; 10 British Heart Valve Society, Swaffham, UK; 11 North West Anglia Foundation Trust, Peterborough, UK; 12 Whaddon Healthcare, Milton Keynes, UK; 13 Royal United Hospitals Bath NHS Foundation Trust, Bath, UK; 14 Department for Health, University of Bath, Bath, UK; 15 Oxford University Hospitals NHS Foundation Trust, Oxford, UK

**Keywords:** Transthoracic echocardiography, Echocardiography, Indications, Triage

## Abstract

Transthoracic echocardiography (TTE) is widely utilised within many aspects of clinical practice, as such the demand placed on echocardiography services is ever increasing. In an attempt to provide incremental value for patients and standardise patient care, the British Society of Echocardiography in collaboration with the British Heart Valve Society have devised updated guidance for the indications and triaging of adult TTE requests for TTE services to implement into clinical practice.

## Introduction

Transthoracic echocardiography (TTE) is widely accepted as the first choice non-invasive imaging modality for the assessment of cardiac structure and function. TTE is versatile, widely available and low-cost as such it forms a crucial part for a patient’s pathway throughout clinical practice including initial diagnosis, management and follow-up of many cardiac [1, 2] but also non-cardiac conditions.

The demand on TTE services continues to rise year upon year with data from National Health Service England indicating a ~ 3% increase in TTE activity for 2019 alone [3]. This continued increase in activity will invariably result in delays to patients accessing TTE’s services. As such, the British Society of Echocardiography (BSE) in collaboration with the British Heart Valve Society (BHVS) have written an update to the previous ‘Clinical Indications for Echocardiography’ [4] in an attempt to assist healthcare professionals in recognising when TTE is indicated and will provide incremental value to the management of a patient. This guide also provides guidance on the implementation of a standardised triaging system for TTE requests. It is thought that together this can achieve a more sustainable service for the benefit of our patients.

This guideline is based on evidence from relevant clinical studies and/or general agreement from clinical practice. It is intended to be used by all BSE members throughout the United Kingdom. It will be regularly reviewed (5 yearly) and updated in accordance with changes as directed by publications and/or changes in clinical practice as and when required. This guideline is not intended to include recommendations specifically relating to the practice of transoesophageal or stress echocardiography. Occasionally, attention may be drawn to circumstances where utilisation of an alternative cardiac imaging modality such as cardiac computed tomography (cCT) or cardiac magnetic resonance imaging (cMRI) may be preferable, this will be highlighted where appropriate.

It is important to recognise that although there is a need to expand and diversify TTE services, it remains imperative that a well performed TTE, undertaken by an appropriately trained healthcare professional, is essential to ensure a cost effective, high quality and sustainable TTE service [2]. BSE do not support the implementation of level I or focussed TTE studies. Furthermore, BSE do not support the use of non-accredited healthcare professionals  to facilitate the reduction in waiting lists. The BSE has published practical guidance on the minimum dataset recommended for a standard adult TTE [5] and these should be implemented in practice.

## Incorporating clinical assessment by telephone into triage

**Fig. 1:**
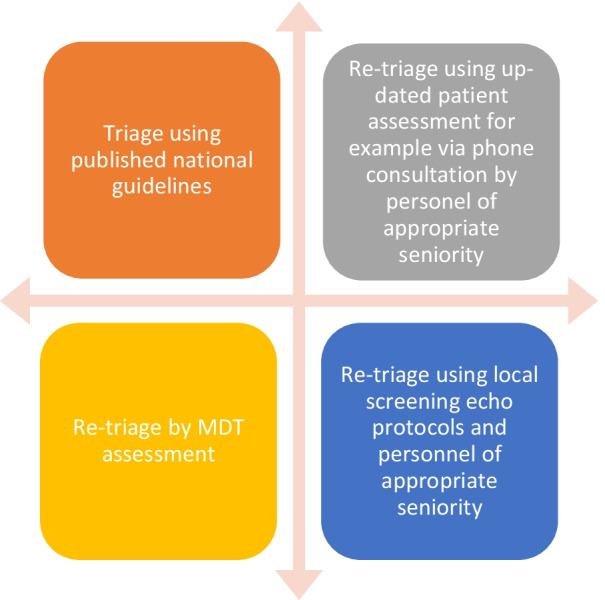
Model for the effective triage of existing (long-standing) and new transthoracic echocardiography requests by appropriately trained clinical specialists only and in-sight of the patients medical records

**Fig. 2:**
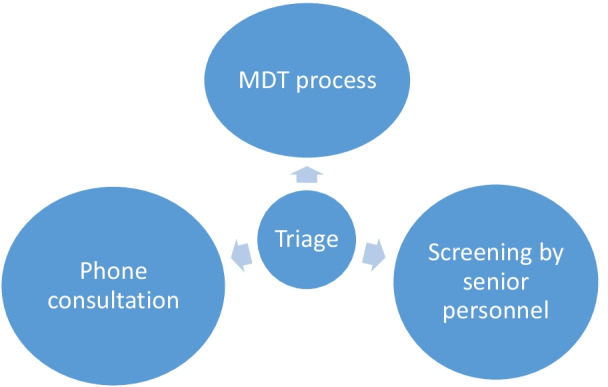
Escalation or interaction of triage processes for more complex referrals

For long-standing TTE referrals, individual services may wish to utilise appropriately trained clinical colleagues (Cardiology Consultant/Specialist Registrar, Cardiac Scientist or Specialist Cardiac Nurse) to re-contact patients to ascertain the clinical appropriateness and/or urgency of TTE request. The content of these discussions will be bespoke to each individual patient and should only be undertaken with sight of a patient’s medical records. This process may also be guided by locally available resources as outlined in Figs. 1 and 2. In light of these complex dependencies, triage using clinical assessment by telephone can be undertaken for follow-up requests if needed whilst acknowledging that it may be difficult to grade symptoms or undertake a clinical examination remotely. Furthermore, where follow-up periods of tests are lengthened, patients should be provided with the necessary information required to reconnect with the appropriate teams should there be a change in symptoms. It is vital that this is communicated sensitively to the patient explaining the rationale behind these decision. Any change in the patient’s clinical status should prompt a thorough reassessment.

## Clinical indications and triage of echocardiography

Appropriateness criteria for TTE will be classified with respect to the sub-headings below.

- Out-patient requests
- Ward based and high dependency inpatients
- Follow-up requests

Didactic timeframes for the delivery of echocardiography services are inherently difficult and this decision-making process will require careful balance with additional clinical pressures on staff. As a broad outline, if an appropriate outpatient TTE is received and is triaged as “indicated” this should be undertaken within a six week time frame. For TTE requests that are triaged as “urgent” this should be undertaken within a two week time frame (unless otherwise indicated in Table 1). The list provided is not exhaustive but is designed to provide a framework for departments to streamline service provision whilst giving clear guidance to referring healthcare professionals for when TTE is appropriate. It is advocated that sufficient time is devoted to accurate triage; national experience suggests that a clinical focus on triage is able to increase capacity for TTE delivery.

**Table 1:** Appropriateness, timing and triage of the most common outpatient TTE referrals

**1. Heart murmur** [6]		
***Not indicated:*** • Assessment of an innocent murmur*diagnosed by a physician • Unchanged murmur in an asymptomatic individual with previous normal echocardiogram	***Indicated:*** • Murmur in the presence of cardiac or respiratory symptoms • Murmur in an asymptomatic individual in whom clinical features or other investigation suggest structural heart disease	***Urgent:*** • Murmur in the presence of class 3 or 4 heart failure symptoms or syncope
*An innocent murmur has previously been defined as: A systolic murmur of short duration, grade 1 or 2 intensity at the left sternal border, a systolic ejection pattern, a normal S2, no other abnormal sounds or murmurs, no evidence of ventricular hypertrophy or dilation, no thrills, and the absence of an increase in intensity with the Valsalva manoeuvre. Such murmurs are especially common in high-output states such as pregnancy [7, 8]		
**2. Suspected heart failure**		
***Not indicated:*** • Radiographic cardiomegaly with no symptoms or signs of heart failure and in the absence of other clinical information • Assessment of patients with peripheral oedema but normal jugular venous pressure and no evidence of cardiac disease (e.g., asymptomatic with a normal 12 lead ECG) • Patients in AF with an uncontrolled ventricular rate (unless class 3 or 4 heart failure symptoms)	***Indicated:*** • Clinical signs of heart failure (e.g., peripheral oedema, bilateral pleural effusions) • Unexplained shortness of breath in the absence of clinical signs of heart failure if ECG/CXR abnormal • Persistent hypotension of unknown cause • Suspected cardiomyopathy based on abnormal examination, ECG, or family history in first degree relative • Assessment of neuromuscular diseases associated with cardiac manifestations, (e.g., muscular dystrophies, Friedreich's ataxia or mitochondrial myopathies)	***Urgent:*** • Class 3 or 4 heart failure symptoms • Raised NT-pro BNP* or previous history of myocardial infarction • Clinical suspicion of pericardial effusion • *NT-proBNP* > *2000 ng/litre requires TTE within 2 weeks. NTproBNP between 400 and 2000 ng/litre requires TTE within 6 week* [9]
**3. Hypertension and suspected left ventricular hypertrophy**		
***Not indicated:*** • Routine assessment of any patient with essential hypertension • Routine assessment of asymptomatic patients with an established genetic or infiltrative cause of LVH where there is no change in clinical status and where an echo has been performed within the last 12 months • Repeat assessment of LV function in asymptomatic patients • Repeat assessment for left ventricular mass regression (if clinical concern is present regarding hypertrophic cardiomyopathy then repeat assessment with cMRI is preferable)	***Indicated:*** • Suspected LV dysfunction • Evaluation of clinically suspected aortic co-arctation (e.g., hypertension in the young) • Elevated blood pressure with concerns for end organ damage • Patients with a suspected or established genetic or infiltrative cause of LVH (with support from appropriate specialist teams where relevant)	***Urgent:*** • Accelerated hypertension with breathlessness or other clinical concerns of acute LV dysfunction
**4. Suspected cardiac mass/possible cardiac cause of systemic-circulation embolism**		
***Not indicated:*** • Patients with terminal illness whose management would not be affected by identification of any TTE abnormalities • Patients in whom TTE will not affect the decision to commence anticoagulation (e.g., patients in AF with cerebrovascular event and no suspicion of structural heart disease)	***Indicated:*** • Embolic peripheral or neurological events suggesting intracardiac mass: ◦ Acute interruption of blood flow to major peripheral or visceral artery ◦ Unexplained stroke or TIA without evidence of prior cerebrovascular disease or without significant risk factors for other cause (consider saline-contrast echocardiography by TTE or TOE, this may only be appropriate in < 55 year old patients) • Cross-sectional imaging or clinical findings suggesting intra-cardiac mass (if possible left atrial appendage thrombus then TOE preferable) • Periodic repeat assessment following removal of cardiac mass/tumour (usually annual review will suffice after an initial post-op scan) • Known primary malignancies where echocardiographic surveillance for cardiac involvement forms part of the normal staging process (e.g., renal cell carcinoma)	***Urgent:*** • Embolic event in the presence of clinical or ECG suspicion of significant left ventricular impairment (e.g., anterior Q waves on 12 lead ECG or clinical examination findings suggestive of LV dysfunction)
**5. Pulmonary disease**		
***Not indicated:*** • Repeat assessment to evaluate the probability of PHT in the absence of a meaningful tricuspid regurgitation jet or other echo markers of PHT on echo within the last 12 months. If there is clinical concern regarding PHT then advice from a pulmonary hypertension specialist service is recommended • Lung disease with no clinical suspicion of cardiac involvement or PHT	***Indicated:*** • Lung disease combined with a clinical suspicion of RV failure (e.g., peripheral oedema, raised jugular venous pressure) • Following pulmonary embolism when clinical concern for right ventricular impairment and/or presence of developing PHT • Evaluation for suspected or established PHT • Evaluation of response to treatment of PHT and PE • To distinguish cardiac from non-cardiac causes of dyspnoea when the results of clinical and other diagnostic testing are ambiguous • Patients with unexplained persistent or positional oxygen desaturation (consider bubble-contrast echocardiography to evaluate for a right to left shunt)	***Urgent:*** • Not applicable
**6. Prior to cardioversion in patients with atrial fibrillation**		
***Not indicated:*** • Patients requiring emergency cardioversion • Patients on long-term anti-coagulation at a therapeutic level with no clinical suspicion of structural heart disease • Patients on long-term anti-coagulation at a therapeutic level with structural heart disease but no recent clinical change	***Indicated:*** • To guide decision-making regarding DC cardioversion in a patient with no recent echo study (i.e. within the last 12 months) or in a patient with a recent echo study and a change in clinical cardiovascular status since it was performed • Patients requiring cardioversion with AF of greater than 48 h duration and not adequately anticoagulated (TOE required) • Repeat assessment of documented left atrial appendage thrombus (TOE required) Repeat assessment following an embolic event at previous cardioversion (TOE required) • Patients with AF of less than 48 h duration together with a clinical suspicion of structural heart disease and not adequately anticoagulated (TOE required)	***Urgent:*** • Not applicable
**7. Palpitations and pre-syncope/syncope**		
***Not indicated:*** • Palpitations without ECG proof of arrhythmia or clinical suspicion of structural heart disease on examination • Low-burden (< 5%) or isolated ventricular ectopy in absence of a clinical suspicion of structural heart disease • Classic neuro-cardiogenic syncope	***Indicated:*** • Clinical suspicion of structural heart disease in proven arrhythmia (e.g., AF or ventricular ectopy at greater than 10% frequency or ventricular ectopy occurring on exertion) • Routine assessment of ventricular function to assist with the calculation of risk of sudden cardiac death post-myocardial infarction or following documented ventricular tachycardia • Evaluation of cardiac structure and function to assist with future management (e.g., commencement of anti-arrhythmic medications) • Syncope in a patient with high-risk occupation (e.g., pilot, bus driver) • Assessment of patients without clinical suspicion of structural heart disease who have an arrhythmia commonly associated with structural heart disease (e.g., ventricular tachycardia)	***Urgent:*** • Syncope in a patient with clinically suspected structural of functional heart disease • Exertional syncope
**8. Suspected pericardial disease**		
***Not indicated:*** • Repeat assessment of small pericardial effusion (< 1 cm) with no hemodynamic compromise and without a change in clinical status • Follow-up studies in patients with terminal illness whose management would not be affected by echocardiographic abnormalities	***Indicated:*** • Clinically suspected pericarditis, pericardial effusion, or pericardial constriction • Periodic repeat assessment of moderate or large pericardial effusion • Repeat assessment of small pericardial effusion with change in clinical status	***Urgent:*** • Clinical suspicion of cardiac tamponade (especially if predisposing factors are present, e.g., known malignancy, suspected myo-pericarditis and recent cardiac surgery)
**9. Established cardiomyopathy**		
***Not indicated:*** • Patients with terminal illness whose management would not be affected by identification of any change in TTE appearance • Routine repeat assessment in clinically stable patients in whom no change in management is contemplated	***Indicated:*** • Repeat assessment in documented cardiomyopathy where the result may change management or following procedures that may improve ventricular function (e.g., cardioversion or coronary revascularisation) • Repeat assessment in documented cardiomyopathy where there has been a change in clinical status	***Urgent:*** • New onset class 3 or 4 heart failure symptoms
**10. Aortopathy**		
***Not indicated:*** • Patients with terminal illness whose management would not be affected by identification of any change in TTE appearance	***Indicated:*** • Assessment of suspected or proven genetic disorders in which aortic pathology may be a feature, (e.g., Marfan Syndrome) • Diagnosis and periodic assessment of aortic aneurysm, dilatation of the aorta and previous surgical repair of the aorta (an annual default interval between scans, but this timeline may be superseded following multi-disciplinary team review). Due to the limited ability of TTE to visualise the thoracic aorta the appropriate concomitant use of cross-sectional imaging is recommended	***Urgent:*** • Clinical suspicion of an acute aortic event (should not replace or delay cross-sectional imaging if more clinically appropriate)
**11. Elective non-cardiac surgery**		
***Not indicated:*** • Routine pre-operative assessment • Repeat assessment of previous echocardiogram in last 12 months with no intervening change in clinical status	***Indicated:*** • Murmur in an asymptomatic individual in whom clinical features suggest severe structural heart disease • Documented ischemic heart disease with reduced functional capacity (< 4 METs) • Murmur in the presence of cardiac or respiratory symptoms	***Urgent:*** • Not applicable

*AF (Atrial fibrillation), ECG (electro-cardiogram), NT-pro BNP (N-terminal pro hormone brain natriuretic peptide), CXR (chest x-ray), LV (left ventricular) TOE (trans-oesophageal echocardiogram), TTE (trans-thoracic echocardiogram), PHT (pulmonary hypertension), DC (direct current)*

## Out-patient requests

A complete and comprehensive overview of appropriate TTE indications can be seen in Table 1. Out-patient requests have been broadly divided into those most often referred for TTE. These include:

- Heart murmur.
- Suspected heart failure.
- Hypertension and suspected left ventricular hypertrophy.
- Suspected cardiac mass/possible cardiac cause of systemic-circulation embolism.
- Pulmonary disease.
- Pre-cardioversion in patients with atrial fibrillation.
- Palpitations and pre-syncope/syncope.
- Suspected pericardial disease.
- Established cardiomyopathies.
- Aortopathy.
- Elective non-cardiac surgery.

These ‘most common’ referral indications have also undergone further sub-classification to determine appropriateness and urgency.

1. **‘*Not indicated’*** TTE is unlikely to offer significant value to patient management and the request should therefore be declined.
2. ***‘Indicated’*** TTE is appropriate and should be completed routinely.
3. ***‘Urgent’*** TTE is appropriate and should be undertaken as a clinical priority.

It is acknowledged that patients with suspected heart failure may present with a variety of clinical signs and symptoms. Suspected heart failure referrals that are described as “urgent” are consistent with NICE guideline [9]. Suspected heart failure referrals that are described as “indicated” are an attempt to include a broader range of clinical signs and symptoms where there is likely to be a high diagnostic yield for abnormality. Suspected heart failure referrals that are described as “not-indicated” are an attempt to reduce the rate of inappropriate referrals that are likely to yield a low level of abnormality whilst ensuring departments receive appropriate requests with pertinent details.

## Ward based in-patient requests

The process for the handling of routine, urgent and emergency inpatient TTE referrals has been published previously [10]. This comprises the following indications and should be subject to immediate triage.

Clinical suspicion of:

- Circulatory failure due to hypovolaemia.
- Acute decompensation in cardiac function.
- Acute or severe valve pathology: critical aortic stenosis/regurgitation or mitral valve dysfunction.
- Acute right heart impairment due to pulmonary embolus.
- Cardiac tamponade.

In addition to the above TTE guidance, the following indications and triage categories are specific to an inpatient setting (see Table 2). Where appropriate, inpatient TTE referrals are sub-classified with respect to urgency and subsequent change in clinical management of a given patient.

1. ***‘Not indicated’*** TTE is unlikely to offer significant value to patient management and the request should be declined.
2. ***‘Indicated’*** TTE is appropriate and should be completed as a non-urgent inpatient. Here, the timeframe is variable and will depend upon clinical need.
3. ***‘Urgent’*** TTE is appropriate and should be undertaken within 24 h.
4. ***‘Emergency’*** TTE is appropriate and should be undertaken within 60 min.

**Table 2:** Appropriateness, timing and triage of the most common inpatient TTE referrals

**1. Chest pain**			
***Not indicated:***	***Indicated:***	***Urgent:***	***Emergency:***
• Evaluation of cardiac chest pain with a normal ECG, no murmur and negative cardiac biomarkers	• Following confirmed acute myocardial infarction to assess for infarct size and complications	• Murmur following a recent acute myocardial infarction	• Chest pain with haemodynamic instability • Assessment of suspected type I aortic dissection often in conjunction with cross-sectional imaging
**2. Suspected heart failure**			
***Not indicated:***	***Indicated:***	***Urgent:***	***Emergency:***
• Not applicable	• Not applicable	• Patients admitted for suspected heart failure commenced on inpatient treatment	• Cardiogenic shock as judged by an appropriately senior clinician • Return of circulation following cardiac arrest
**3. Syncope**			
***Not indicated:***	***Indicated:***	***Urgent:***	***Emergency:***
• No murmur detected or documented malignant arrhythmias. Vaso-vagal or situational syncope	• Not applicable	• Murmur detected clinically • Arrhythmia-associated syncope • Significantly abnormal ECG e.g., LBBB, RBB or LVH	• Not applicable
**4. Arrhythmias**			
***Not indicated:***	***Indicated:***	***Urgent:***	***Emergency:***
• Fast AF without hypotension or suspicion of structural heart disease • Symptomatic ectopics (defer to outpatient following Holter monitoring)	• Not applicable	• Arrythmia and hypotension • Ventricular tachycardia or ventricular fibrillation • Clinical suspicion of infective endocarditis with evidence of acute cardiac failure, valve decompensation, or abscess	• Not applicable
**5. Suspected or established pulmonary embolism** [11]			
***Not indicated:***	***Indicated:***	***Urgent:***	***Emergency:***
• Asymptomatic patient post therapy for a CTPA confirmed PE and/or right heart strain (defer to 3 month OP TTE) • Pre-discharge to evaluate for features of persisting right heart strain in clinically stable patients (defer to 3 month OP echo)	• Re-evaluation for further therapy where CV compromise does not resolve with treatment	• To establish right heart function in clinically unstable patients to facilitate decision making regarding thrombolysis or alternative therapies	• Not applicable
**6. Emergency non-cardiac surgery**			
***Not indicated:***	***Indicated:***	***Urgent:***	***Emergency:***
• Known ventricular or valvular dysfunction established on TTE within 12 months without a change in symptoms • AF without signs of congestive cardiac failure or murmur • Referral based on age or frailty only	• Not applicable	• Clinical suspicion of significant valvular or ventricular pathology which will alter the anaesthetic approach (e.g., LBBB, RBBB or significant LVH)	• Not applicable
**7. Infective endocarditis**			
***Not indicated:***	***Indicated:***	***Urgent:***	***Emergency:***
• Fever with no other positive Duke criteria • Repeat assessment in a clinically stable patient with known vegetations	• To characterise valve lesions and haemodynamic consequences where Duke’s criteria are positive • One week following a negative TTE study in cases of high clinical suspicion where a TOE is not possible • Detection of high-risk complications when suspected (e.g., fistula, abscess, mass lesions) • Persistent bacteraemia of unknown source, particularly in staphylococcal aureus infection • Baseline re-assessment prior to discharge following completion of treatment for endocarditis	• Not applicable	• Not applicable
**8. Post cardiac operation or procedure**			
***Not indicated:***	***Indicated:***	***Urgent:***	***Emergency:***
• Following routine elective coronary revascularisation in stable patients • Routine pre-discharge echo following valve replacement in asymptomatic patients. Obtain baseline haemodynamic data at 4–6 weeks post operation	• Routinely following AF ablation • Routinely following structural heart disease intervention e.g., PFO closure	• Concern regarding cardiac tamponade following any cardiac or thoracic cavity procedure • Concern regarding cardiac tamponade following structural heart disease procedure, coronary intervention or permanent/temporary pace-maker insertion or lead extraction	• Not applicable
**9. Acute stroke**			
***Not indicated:***	***Indicated:***	***Urgent:***	***Emergency:***
• Patient not in AF with no murmurs or suspicion of regional wall motion abnormality	• Patient in AF • Audible murmur • Suspected regional wall motion abnormality from clinical assessment or ECG	• Not applicable	• Not applicable
**10. Specific indications for TTE** [14] Shock: TTE is recommended as the primary assessment tool for the shock state following senior clinical assessment			
***Not indicated:***	***Indicated:***	***Urgent:***	***Emergency:***
• Prior to clinical assessment and initial management	• Not applicable	• Where initial clinical assessment and management has failed to provide reasonable clinical improvement	• Not applicable
**11. Assessment of right heart function (see prior section for pulmonary embolism)**			
***Not Indicated:***	***Indicated:***	***Urgent:***	***Emergency:***
• Not applicable	• Not applicable	• Where acute right heart dysfunction is clinically suspected (e.g., due to the use of high positive end expiratory pressure ventilation strategy or where ECG changes suggest right ventricular infarction)	• Not applicable
**12. Assessment of left ventricular function**			
***Not indicated:***	***Indicated:***	***Urgent:***	***Emergency:***
• Where clinical information is otherwise adequate to answer the clinical question	• Not applicable	• Following cardiac arrest and return of circulation • In cases of severe malnutrition • Where underlying cardiomyopathy is suspected	• Where there is difficulty in maintaining end organ perfusion despite senior assessment and therapy • Where a direct effect of pathology on ventricular function is suspected e.g., septic cardiomyopathy
**13. Assessment of complex fluid balance**			
***Not indicated:***	***Indicated:***	***Urgent:***	***Emergency:***
• Prior to clinical assessment and initial management	• Not applicable	• To determine filling status in anuric state • To guide renal replacement therapy and fluid therapy planning	• Where despite evidence to the contrary hypovolaemia may be the cause of hypotension/perfusion e.g., following large volume resuscitation or where peripheral oedema is present
**14. Differentiation between acute respiratory distress syndrome and pulmonary oedema**			
***Not indicated:***	***Indicated:***	***Urgent:***	***Emergency:***
• Where the cause of interstitial fluid appearance on chest radiology is known for example in acute pneumonitis diagnosed on cCT imaging	• Not applicable	• Not applicable	• Where there is reasonable clinical suspicion that the cause of interstitial fluid seen on chest radiography or lung ultrasound is due to raised left ventricular end diastolic pressure
**15. Suspicion of acute mechanical valvular pathology**			
***Not indicated:***	***Indicated:***	***Urgent:***	***Emergency:***
• Where history examination and current illness are not supportive of a diagnosis of valve dysfunction as a cause for haemodynamic compromise	• Not applicable	• Where the history and examination findings suggest that the clinical picture and/or organ failure may be due to critical or acute valve dysfunction, e.g., flail mitral valve	• Not applicable
**16. Assessment of the pericardial space**			
***Not indicated:***	***Indicated:***	***Urgent:***	***Emergency:***
• Small volume pericardial effusion is noted on cCT in the context of critical illness without haemodynamic effects	• Not applicable	• Where there is clinical suspicion of pyopericardium from clinical, microbiological and radiological information	• Where clinical findings suggest that known or suspected pericardial fluid is either contributing to haemodynamic compromise or causing acute cardiac tamponade
**17. Special circumstances**			
***Not indicated:***	***Indicated:***	***Urgent:***	***Emergency:***
• Not applicable	• Assessment of cardiac function to facilitate organ donation • Guidance for positioning of extracorporeal support cannulae • Search for penetrating objects or assessment of cardiac structure following trauma to the thorax	• Not applicable	• Not applicable

*AF (atrial fibrillation), cCT (computed tomography), CTPA (computed tomography pulmonary artery), CXR (chest x-ray), DC (direct current), ECG (electro-cardiogram), LBBB (left bundle branch block), LVH (left ventricular hypertrophy), OP (out-patient), PFO (patent foramen ovale), PE (pulmonary embolus), PHT (pulmonary hypertension), RBBB (right bundle branch block), RV (right ventricle), TOE (trans-oesophageal echocardiogram), TTE (trans-thoracic echocardiogram)*

It is acknowledged that service design and capabilities will vary between centres and the above time frames are given as optimum targets.

## Follow-up requests

For those patients requiring longer term TTE follow-up, effective triage of referrals is vitally important to ensure resources are allocated to those with the most appropriate clinical need. Local clinical pathways, staffing and specialist skill-sets will, in some part, dictate appropriateness and timing of follow-up in these patients. However, this guidance advocates triage of referrals by experienced Healthcare Professionals to ensure clinical needs are met and an effective service is delivered.

TTE follow-up has been sub-classified into native valve disease (see Tables 3, 4 and 5) and prosthetic valves, valve repair and aortic disease (see Table 6). Latest NICE guidance [6] advocates the use of BSE guidelines for the assessment of valve disease severity including aortic stenosis [17], tricuspid and pulmonary valve disease [18] and mitral valve disease [19]. The included follow-up timeframes for native valve disease, valve replacement, valve repair and aortic diseases allow for standardisation of care and are in keeping with previously published literature [13–16]. The incorporation of “echo alerts” and “other alerts” are designed to highlight TTE features that should prompt urgent specialist review. There are no clinically significant differences between the recommendations herein and those published by the European Society of Cardiology [20] and the American College of Cardiology [21]. Patients under the care of a valve clinic and who require “Cardiologist / urgent Cardiologist review” should be undertaken by a valve clinic Cardiologist with valve disease competencies and expertise as this promotes best practice [22].

**Table 3:** Native valve follow-up: aortic stenosis and aortic regurgitation*

Aortic stenosis		
**Mild**	**Moderate**	**Severe**
• Vmax: 2.6–2.9 m/s: TTE every 3–5 years • Cardiologist review if symptomatic	• Vmax: 3.5–3.9 m/s: TTE every 12–18 months • Vmax: 3.0–3.4 m/s: TTE every 18–24 months • Valve area: 1.0–1.5cm^2^ • Cardiologist review	• Vmax: > 4 m/s • Aortic valve area: < 1 cm^2^ • TTE every 6 months • Cardiologist review
**TTE alerts for urgent Cardiologist review:** • LVEF < 50% or reduced flow • Cardiology discussion advised if severe AS and reducing LVEF on sequential TTE’s, LVEF in range of 50–60% • Rapid progression of Vmax: > 0.3 m/s per year • Dilated aortic root (≥ 45 mm In Marfans syndrome; ≥ 50 mm in bicuspid aortic valve patients; ≥ 55 mm for all other patients		
**Other alerts for Cardiologist review advised:** • Development of symptoms: breathlessness, chest pain, pre-syncope, syncope **TTE follow-up for bicuspid aortic valves:** • Bicuspid valve with no stenosis and mild regurgitation: TTE every 3–5 years • Bicuspid valve with thickening and mild stenosis: TTE every 2 years **Aortic sclerosis:** • Valve thickening and peak velocity ≤ 2.5 m/s: TTE every 5 years (no follow-up usually needed in > 80 years of age unless restricted cusp excursion)		

Aortic regurgitation		
**Mild**	**Moderate**	**Severe**
• Mild to moderate: TTE every 3–5 years • Cardiologist review if aortic root dilated	• TTE every 1–2 years • Cardiologist review	• TTE every 6–12 months • Cardiologist review
**TTE alerts for urgent Cardiologist review:** • LVEF < 50% • Cardiology discussion advised if severe AR and reducing LVEF on sequential scans, LVEF in range of 50–60% • LV systolic dimension approaching 50 mm; LV diastolic diameter approaching 70 mm or severe LV volume dilatation • Dilated aortic root (≥ 45 mm In Marfans syndrome; ≥ 50 mm in bicuspid aortic valve; ≥ 55 mm for all other patients)		
**Other alerts: Cardiologist review:** • Development of symptoms • Trace-mild AR associated with normal aortic valve morphology, normal aortic root and normal ascending aorta does not usually require TTE surveillance		

*AR (aortic regurgitation), AS (aortic stenosis), cm2 (centimetres squared), LV (left ventricular), LVEF (left ventricular ejection fraction), m/s (meters per second), mm (millimetres), TTE (Transthoracic echocardiography), Vmax (maximum velocity)*

*Adapted from Baumgartner et al. 2017 [13] and Chambers et al. 2017 [14]

**Table 4:** Native valve follow-up: mitral stenosis and mitral regurgitation*

Mitral stenosis		
**Mild**	**Moderate**	**Severe**
• Valve area > 1.5 cm^2^ • TTE every 3–5 years	• Valve area: 1.0–1.5 cm^2^ • TTE every 1–2 years • Cardiologist review	• Valve area: < 1.0 cm^2^ • TTE every 6–12 months • Cardiologist review
N.B. Valve area < 1.5 cm^2^ = clinically significant mitral stenosis where valve intervention can be considered if patient is symptomatic or, asymptomatic with high risk of embolism/decompensation or, positive exercise stress echocardiography		
**TTE alerts for urgent Cardiologist review:** • PA systolic pressure > 50 mmHg • RV dysfunction • Dense spontaneous contrast in the LA		
**Other alerts for Cardiologist review:** • Development of symptoms • New AF • TIA or stroke		

Mitral regurgitation		
**Mild**	**Moderate**	**Severe**
• TTE every 3–5 years if mild prolapse • No follow up usually required if normal mitral valve appearance	• TTE every 1–2 years • Cardiologist review	• TTE every 6–12 months • Cardiologist review at 6 months
**TTE alerts for urgent Cardiologist review:** • LVEF < 60% • LV systolic dimension approaching 45 mm • Severe LV volume dilatation • MR secondary to flail leaflet • PA systolic pressure > 50 mmHg • Severe MR with LA volume ≥ 60 ml/m^2^ and patient in sinus rhythm		
**Other alerts: Cardiologist review:** • Development of symptoms • New AF		

*AF (atrial fibrillation), AR (aortic regurgitation), AS (aortic stenosis), cm2 (centimetres squared), LA (left atrial), LV (left ventricular), LVEF (left ventricular ejection fraction), mm (millimetres), MR (mitral regurgitation), m/s (meters per second), PA: pulmonary artery, RV (right ventricular),TIA (transient ischemia attack), TTE (Transthoracic echocardiography), Vmax (maximum velocity)*

*Adapted from Baumgartner et al. 2017 [13] and Chambers et al. 2017 [14]

**T﻿able 5:** Native valve follow-up: right heart valvular pathology (pulmonary/tricuspid regurgitation)*

Pulmonary/tricuspid regurgitation		
**Mild**	**Moderate**	**Severe**
• No follow up usually needed if mild or moderate TR and normal valve and normal RV	• With abnormal valve or RV: • TTE every 2 years • Cardiologist review	• TTE every year • Cardiologist review
**TTE alerts for urgent Cardiologist review:** • RV dysfunction • RV dilatation		
**Other alerts for Cardiologist review:** • Development of symptoms: breathlessness, chest pain, pre-syncope, syncope		

Pulmonary stenosis		
**Mild**	**Moderate**	**Severe**
• Vmax: < 2 m/s • TTE every 3–5 years	• Vmax: 3.0–4.0 m/s • TTE every 2 years • Cardiologist review	• Vmax: > 4 m/s • TTE every year • Cardiologist review
**TTE alerts for urgent Cardiologist review:** • RV dysfunction • RV dilatation		
**Other alerts for Cardiologist review:** • Development of symptoms		

*m/s (meters per second), RV (right ventricular), TTE (transthoracic echocardiography), TR (Tricuspid regurgitation), Vmax (maximum velocity)*

*Adapted from Baumgartner et al. 2017 [13] and Chambers et al. 2017 [14]

**Table 6:** Follow-up: prosthetic valve replacement, valve repair and aorta*

Valve replacement/repair			
**Mechanical valve replacement**	**Biological valve replacement (surgical)**		**Mitral valve repair (surgical)**
• Baseline (4–6 weeks post operatively) • If baseline TTE normal and no alerts (see below), no routine surveillance • If regurgitation review by native valve criteria; consider TOE	• Baseline (4–6 weeks post operatively) • MV/TV or AV < 60 years (unless alerts, see below): Annual TTE from 5 years post implant (for new valve with no durability data annual from implantation) • AV > 60 years and AV with proven longevity: Annual surveillance TTE from 10 years post implant (unless alerts, see below) If regurgitation review by native valve criteria		• Competent: Baseline (4–6 weeks post operatively), 1 year post op, then every 2–3 years • Incompetent: Individualised plan
**Alerts for TTE/ Cardiologist review:** • New or worsening prosthetic valve regurgitation • Gradient/effective orifice area outside of expected parameters • New LV dilatation or LV systolic dysfunction • Aortic root dilatation. Urgent if ≥ 45 mm in Marfans syndrome; ≥ 50 mm in bicuspid aortic valve; ≥ 55 mm for all other patients • Suggestion of infective endocarditis or previously medically treated prosthetic valve endocarditis • Worsening symptoms or other sonographer concerns			
**Aortic valve and aortic root replacement**		**Aortic root surveillance post bicuspid aortic valve surgery**	
• Ongoing assessment of the aortic root: individualised plan based on clinical, anatomical and surgical features • Reasonable default: 2 yearly cross sectional imaging		• If normal diameter by TTE, then TTE every 3–5 years • If aortic dilatation: individualised plan based on degree of dilatation and rate of progression on sequential TTE • If dilatation on TTE not reproducible with cCT/cMRI (> 2 mm difference): interval imaging with CR or MRI	
**TAVI**		**Mitral valve repair (transcatheter)**	
• Baseline (4–6 weeks post operatively) or as directed by operator, then annular surveillance. If stable then increase TTE surveillance to 2 years • If paravalvular/transvalvular regurgitation review by native valve criteria • If other native valve stenosis/regurgitation, review by native valve criteria • Complex cases with LV dysfunction or multi-native valve disease: individualised plan		• Competent: Baseline (4–6 weeks post operatively), then annually • Incompetent: individualised plan	

*AV (aortic valve), cCT (cardiac computer tomography), cMRI (cardiac magnetic resonance), LV (left ventricular), mm (millimetres), MV (mitral valve), TAVI (transcatheter aortic valve implantation), TTE (transthoracic echocardiography), TV (tricuspid valve), TOE (trans-oesophageal echocardiography)*

*Adapted from Chambers et al. 2019 [16] and Borger et al. 2018 [15]

It is also acknowledged that a considerable number of patients will have more complex disease (e.g., moderate to severe multi-valve disease or post-operative regurgitation with ventricular dysfunction). For these patients, an individualised approach is essential and discussion amongst the clinical team to advise on the surveillance scan period is recommended.

Cardio-oncology is a complex clinical field and established local pathways are encouraged. Discussion amongst key stakeholders is highly advised, and in certain situations it may be considered appropriate to extend out the interval between repeat TTE. However, where there is clinical deterioration with likely cardiac involvement, a shortened time interval between repeat TTE’s may be more appropriate. It is advocated that baseline left ventricular systolic function assessment prior to the use of potentially cardiotoxic chemotherapy agents is triaged and prioritised as an urgent request to prevent delays to the commencement of treatment. A joint BSE and British Cardio-Oncology Society guideline on the TTE assessment of adult cancer patients receiving anthracycline therapy has previously been published [23].

## Implementation of new indications and triage guidelines into real world practice

To assess the impact of the new indications and triage guidelines on service provision, two clinical service audits were undertaken within an inpatient setting (Craigavon Area Hospital, Southern Health and Social Care Trust) and outpatient valve surveillance clinic setting (University Hospitals of North Midlands). Both clinical service audits were registered with the respective Trust's research and development departments.

### Clinical service audit 1: Craigavon Area Hospital

Craigavon Area Hospital is a District General Hospital in Northern Ireland serving an estimated local population of 241,000. It is commissioned to perform over 10,000 TTE’s annually and has an inpatient bed capacity of 450. In an attempt to reduce inpatient TTE waiting times a service audit was undertaken in March 2021. Inpatient TTE referrals were collated over a two week period and reviewed against the BSE/BHVS indications as outlined in this document. Over this two week time frame 200 referrals were received and audited against the new BSE/NHSE guidance. The audit found that 95% of these referrals were undertaken within the recommended timeframes and 89% within 24 h. When inpatient TTE requests were vetted against the new BSE/NHSE guidance, 44.5% of requests were triaged as “not indicated” for an inpatient basis. This reduction in workload enabled the remaining appropriate inpatient TTE referrals to be completed in a more timely fashion. The audit findings were effectively used as an educational tool to improve the knowledge of appropriate inpatient TTE requests with clinical referring teams. It is hoped that this will translate to fewer inappropriate inpatient TTE’s being received in the future and enable patients with the greatest clinical need to have an inpatient TTE in a timely manner and allow for the freeing up of resources to be more effectively directed elsewhere.

### Clinical service audit 2: University Hospital of North Midlands

The University Hospitals of North Midlands serves an estimated local population of 900,000. The centre has a Physiologist/Scientist led heart valve surveillance clinic which was established over 10 years ago. A clinical service audit was undertaken in November 2020 to better understand the patients’ demographics, valve aetiology/severity and follow-up requirements of the patient currently under its care. At the point of audit, the service incorporated the European Society of Cardiology guidelines for the management of valvular heart disease [13]. The results showed there were 1504 patients within the service, mean age 69 years (range 20–99 years).

The most common native valve aetiology was aortic stenosis (30%) with mitral regurgitation and prosthetic valve replacements (aortic and mitral) accounting for 17% and 46% of patients respectively. This Physiologist/Scientist led service was shown to be safe with low between-appointment non-elective admission rates for patients with moderate/severe disease (5%) and a high proportion of patients referred for valvular intervention (80%) following referral back to medical follow-up when symptoms developed. The release of BSE/NHSE indications for valvular heart disease follow-up, as outlined in this document, prompted an additional audit to be undertaken to assess the impact of this on the follow-up duration of patients within the service.

All patients who attended the clinic in March 2021 were reviewed. A total of 88 patients were included (mean age 70 years, 62% male). Of these, 19.6% of patients’ no longer required routine follow-up due to either, mild aortic or mitral regurgitation with normal valvular structure and function or, mechanical valve replacements with no high risk features on baseline TTE. In 33.9% of patients, follow-up could be extended by an average of 41 months due to the presence of mild regurgitation or stenosis. This audit demonstrates that a portion of patients within heart valve surveillance clinics are potentially having follow-up at shorter time duration than is necessary. This may allow for follow-up frequency to be safely extended therefore increasing additional capacity within the service.

## Conclusion

This updated guideline for TTE indications and triage has been produced to aid healthcare professions who refer patients for TTE ensuring that referrals are appropriate and timely. It also allows TTE services across the United Kingdom to standardise their triaging practice allowing limited resources to be directed to those patients in whom the results will most effectively guide diagnosis, management and/or future follow-up. The clinical audit findings presented demonstrate how these guidelines can be implemented into real world practice and have a positive impact on TTE services.

## Data Availability

Not applicable.

## Abbreviations

AF: Atrial fibrillation
AR: Aortic regurgitation
AS: Aortic stenosis
BSE: British Society of Echocardiography
BHVS: British Heart Valve Society
C-19: COVID-19
cCT: Cardiac computed tomography
cCMR: Cardiac magnetic resonance
CTPA: Computed tomography pulmonary artery
CV: Cardiovascular
CXR: Chest x-ray
DC: Direct current
ECG: Electrocardiogram
LA: Left atrium
LBBB: Left bundle branch block
LV: Left ventricle/left ventricular
LVEF: Left ventricular ejection fraction
LVH: Left ventricular hypertrophy
MDT: Multidisciplinary team
NHSE: National Health Service England
NICE: National Institute of Health Care Excellence
NT-pro BNP: N-terminal pro-hormone brain natriuretic peptide
OP: Outpatient
PA: Pulmonary artery
PE: Pulmonary embolism
PFO: Patent foramen ovale
PHT: Pulmonary hypertension
RBBB: Right bundle branch block
TOE: Transoesophageal echocardiography
RV: Right ventricle/right ventricular
TIA: Transient ischemic attack
TR: Tricuspid regurgitation
TTE: Transthoracic echocardiography
