# Correction: Clinical indications and triaging for adult transthoracic echocardiography: a statement by the British Society of Echocardiography

**DOI:** 10.1186/s44156-022-00014-5

**Published:** 2023-03-09

**Authors:** Sadie Bennett, Martin Stout, Thomas E. Ingram, Keith Pearce, Timothy Griffiths, Simon Duckett, Grant Heatlie, Patrick Thompson, Judith Tweedie, Jo Sopala, Sarah Ritzmann, Kelly Victor, Judith Skipper, Shaun Robinson, Andrew Potter, Daniel X. Augustine, Claire L. Colebourn

**Affiliations:** 1 University Hospitals of North Midlands, Stoke‑on‑Trent, ST4 6QG UK; 2 Manchester University NHS Foundation Trust, Manchester, UK; 3 Shrewsbury and Telford Hospitals NHS Trust, Shrewsbury, UK; 4 Southern Health and Social Care Trust, Craigavon Area Hospital, Portadown, UK; 5 British Society of Echocardiography, London, UK; 6 Doncaster and Bassetlaw Teaching Hospitals, NHS Foundation Trust Doncaster Royal Infirmary, Doncaster, UK; 7 Cleveland Clinic, London, UK; 8 Barking, Havering and Redbridge University Hospitals NHS Trust, London, UK; 9 North West Anglia Foundation Trust, Peterborough, UK; 10 Whaddon Healthcare, Milton Keynes, UK; 11 Royal United Hospitals Bath NHS Foundation Trust, Bath, UK; 12 Department for Health, University of Bath, Bath, UK; 13 Oxford University Hospitals NHS Foundation Trust, Oxford, UK

**Correction: Echo Research & Practice 9:5 (2022)** **https://doi.org/10.1186/s44156-022-00003-8**

The authors wish to clarify that the original title of this article [1] was incorrect, and the revised title (above) does not include reference to collaboration with the British Heart Valve Society. Any mention of British Heart Valve Society involvement within the article is also incorrect and should be ignored. In addition, Dr Benoy Shah has withdrawn from the list of authors. The authorship list is therefore: Sadie Bennett, Martin Stout, Thomas E. Ingram, Keith Pearce, Timothy Griffiths, Simon Duckett, Grant Heatlie, Patrick Thompson, Judith Tweedie, Jo Sopala, Sarah Ritzmann, Kelly Victor, Judith Skipper, Shaun Robinson, Andrew Potter, Daniel X. Augustine and Claire L. Colebourn. Any citation of this article should include these changes. 

The article authors are also making following amendments:

1. Confirmation of the position regarding level I, ‘abridged’ and focussed-repeat echocardiography protocols by adding in the following statement (page 5 line 7):

‘The BSE strive to ensure that patients receive the correct test at the correct time by the most appropriately skilled and qualified staff. This is paramount to ensuring patient safety and the safety and wellbeing of those staff performing these procedures. As such, the use of level I studies [5] should be performed only for the exclusion of immediate life-threatening pathology (to trigger a more advanced level II study or other appropriate test) in an acutely unwell patient, following request from an appropriate attending clinician. Level I echocardiography is not recommended in order to reduce waiting list burden. The use of ‘abbreviated’ or ‘abridged’ echocardiography protocols is also not currently recommended as a tool to reduce waiting list burden. The BSE does support the use of focussed-repeat studies in certain clinical scenarios and only where a recent comprehensive level II dataset has already been acquired as a baseline. These include Herceptin monitoring, acute pathologies where therapy has been given (e.g. thrombolysis), re-assessment of pericardial effusion or re-assessment of endocarditis lesions. The BSE has published practical guidance on the comprehensive level II dataset recommended for a standard adult TTE [6] and these should be implemented in practice’.

2. BSE Level I minimum dataset reference added as number [5]. (page 19, line 26.)

Hindocha R, Garry D, Short N, Ingram T, Steeds R, Colebourn C, Pearce K, Sharma V and the Accreditation and Education Committees of the British Society of Echocardiography. A minimum dataset for a level I echocardiogram. A guideline protocol from the British Society of Echocardiography. Echo Research and Practise 2020: 7(2) G51–G58. https://doi.org/10.1530/ERP-19-0060.

3. Correction of the following typographical errors:
  - Week corrected to weeks (page 22, table 1, column 3, row 29).
  - Marfans corrected to Marfan (page 40, table 3, rows 11 and 28).
  - CR corrected to cCT (page 45, table 6, row 23).

The corrections needed have been updated in this correction article and the original article [1] has been corrected. 
